# Case Report: Pediatric Malignant Atrophic Papulosis With Small Bowel Perforation and Positivity of Anticardiolipin Antibody

**DOI:** 10.3389/fped.2021.764797

**Published:** 2021-12-10

**Authors:** Hai-Qing Wang, Yu Guan, Xiao-Pan Gong, You-Tao Chen, Chao Ji

**Affiliations:** ^1^Department of Dermatology, The First Affiliated Hospital of Fujian Medical University, Fuzhou, China; ^2^Department of Dermatology, Fujian Children's Hospital, Fuzhou, China; ^3^Department of Dermatology, Dermatology Hospital of Fuzhou, Fuzhou, China; ^4^Department of Pediatrics, Fujian Maternity and Child Health Hospital, Fuzhou, China

**Keywords:** malignant atrophic papulosis, Degos disease, bowel perforation, anticardiolipin antibody, pediatric

## Abstract

Malignant atrophic papulosis (MAP) is a life-threatening vasculopathy affecting the skin, gastrointestinal tract, central nervous system, pleural membrane, and pericardium. MAP carries a poor prognosis primarily because of its systemic involvement. It is extremely rare in children. Herein, we report a pediatric case of MAP with small bowel perforation and anticardiolipin antibody positivity.

## Introduction

Malignant atrophic papulosis (MAP), also called Köhlmeier–Degos disease, is a rare microangiopathic disorder characterized by atrophic papules with a porcelain-white center. It most often occurs in middle-aged people and is extremely rare in children. We describe here a 4-year-old boy with MAP and small bowel perforation who was also positive for anticardiolipin antibody.

## Case Report

**Figure 1:**
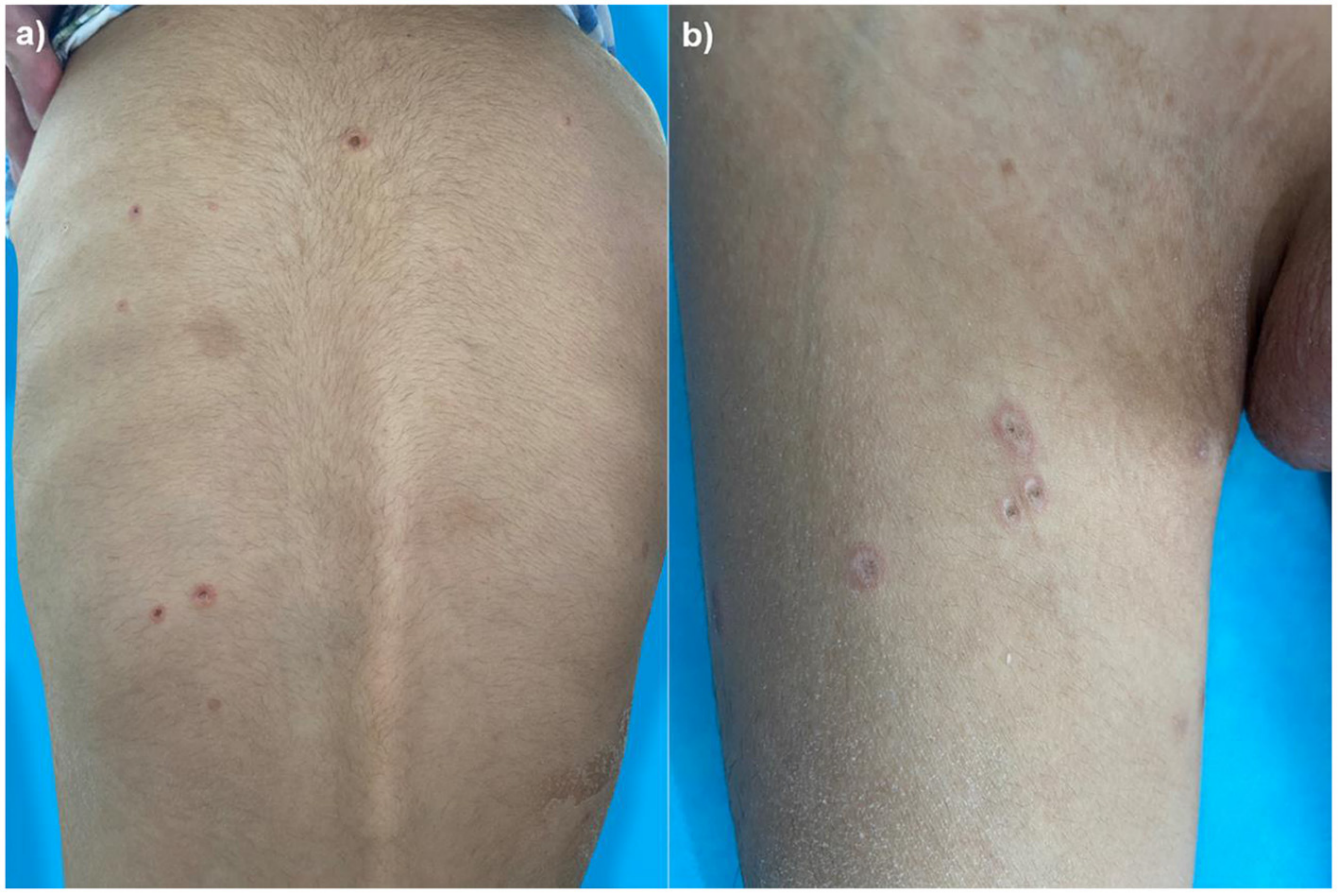
Scarring papules with central ivory depression and rims of erythema on the trunk **(a)** and lower extremities **(b)**.

**Figure 2:**
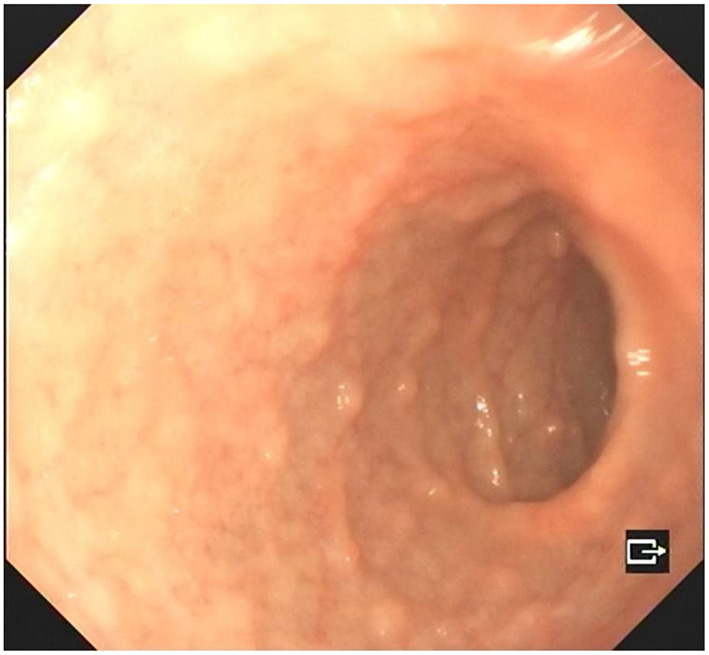
Esophagogastroduodenoscopy showed multifocal raised nodules in the duodenal bulb.

**Figure 3:**
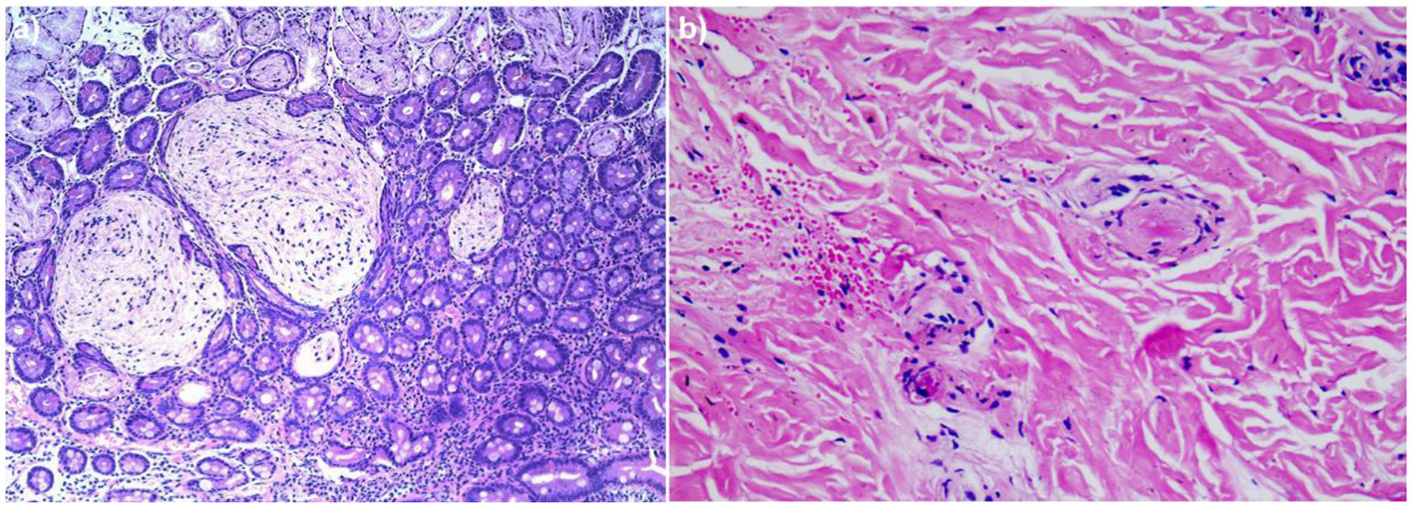
**(a)** Biopsy specimen of duodenal bulb showed focal gland dilatation and inflammatory cells infiltration with focal deposits of mucin (hematoxylin and eosin, ×100). **(b)** Histopathologic examination of the right leg revealed thickened vascular wall with fibrinous necrosis and perivascular lymphocytes infiltration in the dermis (hematoxylin and eosin, ×200).

**Figure 4:**
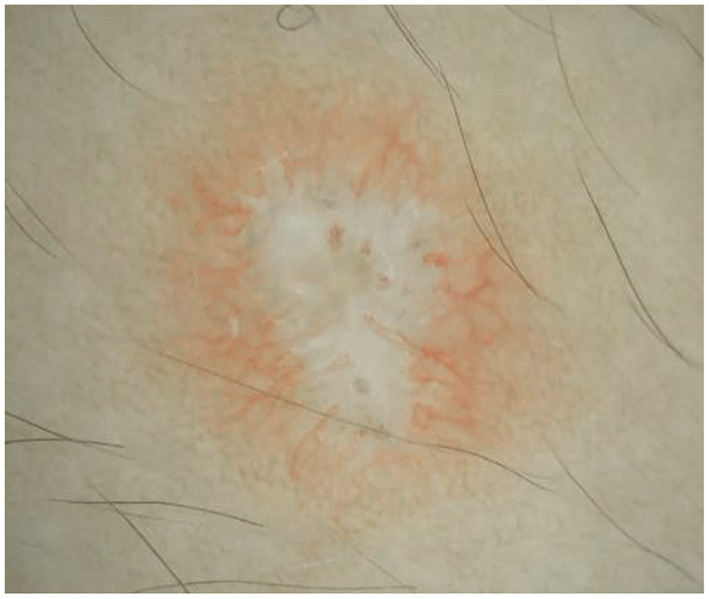
Dermoscopy showed central porcelain-white structureless area crowded by hairpin vessels.

A 4-year-old boy was admitted to our hospital on July 20, 2021, because of recurrent abdominal pain and distention. He had scattered papules with central ivory atrophy or scabs on the trunk, lower extremities, and genital areas. The patient underwent partial resection of the small intestine due to small bowel perforation 1 month prior to hospitalization. At the same time, erythematous papules occurred. One week after the surgery, his abdominal symptoms recurred and worsened over time. Physical examination revealed scattered scarring papules of a mung bean size with central porcelain-white depression over the trunk, lower extremities, and genital areas, sparing the palms and face (Figures 1a,b). An esophagogastroduodenoscopy detected multifocal raised nodules in the duodenal bulb without significant abnormalities in the esophagus, cardia, gastric fundus or corpus, stomach angle, gastric antrum, and pyloric region (Figure 2). And a biopsy of duodenal bulb demonstrated focal gland dilatation and inflammatory cells infiltration with focal deposits of mucin (Figure 3a). Biopsy of a characteristic lesion revealed thickened vascular wall with fibrinous necrosis and perivascular lymphocytes infiltration without mucin deposition in the dermis (Figure 3b). Autoimmune antibodies tests found antinuclear antibody and anticardiolipin antibody were positive. Dermoscopy of atrophic papules revealed a central white structureless area surrounded by hairpin vessels (Figure 4).

Based on these findings, a diagnosis of pediatric MAP was established. The child began treatment with low-dose subcutaneous heparin (5,000 IU/day) and some symptomatic treatments. During the hospitalization, his digestive manifestations were relieved, while the skin lesions only improved slightly. One month later, the patient was lost to follow-up.

## Discussion

MAP, also called Köhlmeier–Degos disease, is an extremely rare vasculopathy that often involves the skin, gastrointestinal tract, central nervous system, pleural membrane, and pericardium (1). The eyes, kidney, and penis can also be occasionally affected (2). Due to the involvement of other organs, the prognosis of MAP is remarkably poor. Gastrointestinal perforation and sepsis are the most common causes of death in patients with MAP (3). The median survival time is 2–3 years, and generally, the average age of disease onset is 20–40 years old (1). Pediatric reports are very rare. We describe here a 4-year-old MAP patient with small bowel perforation and anticardiolipin antibody positivity.

Anti-phospholipid antibodies, including cardiolipin antibodies, bl2-glycoprotein I, and lupus anticoagulant are strongly correlated with autoimmune disorders (2). In 1984, Englert et al. first described the association between Degos disease and antiphospholipid antibodies (4). Thomas Hohwy reported a case of MAP with factor V Leiden mutation and lupus anticoagulant positivity (2). Anti-cardiolipin antibodies have also been implicated in a 26-year-old woman with Degos disease (5). Some experts believe that this phenomenon suggests an association between Degos disease and systemic lupus erythematosus, while others think it might be a random finding of unknown clinical significance since the underlying mechanism remains unclear (2, 6). For this patient, anticardiolipin antibody and antinuclear antibody were both positive but without any specific lesions of connective tissue diseases. So it is most likely that the presence of anticardiolipin antibody is a random finding of MAP. The role of antiphospholipid antibodies on the MAP remains to be elucidated.

The differential diagnosis includes primary antiphospholipid syndrome or the antiphospholipid syndrome resulting from systemic erythematous lupus or other connective tissue diseases (7), such as juvenile dermatomyositis (JDM). In patients with JDM, tissue inflammation is most obvious in the skeletal muscle and skin, presenting symmetric proximal weakness and characteristic lesions. Dermatologic characteristics of JDM comprise the heliotrope rash, Gottron's papules, and nail fold changes (8). In our case, the patient did not have any of these features or muscle weakness. So we did not consider this diagnosis.

It is interesting that High et al. believed MAP may not be a unique entity but may represent a general endpoint to a variety of vascular insults (9). In 2020, Mareschal described MAP as a rare, potentially life-threatening disease classified as a small-vessel vasculopathy (7). Due to the specific clinical presentation and poor prognosis, we consider MAP a specific disease.

The pathogenesis of MAP is uncertain and may involve genetic factors, endothelial cell abnormalities, coagulation dysfunction, vasculitis, and anomalies in complement activation C5b-9 (10, 11). Clinically, MAP is characterized by several to hundreds of pink papules which are surrounded by an erythematous halo and develop a porcelain-white atrophic center (12). On hematoxylin–eosin stain, MAP shows a wedge-shaped area of ischemia, but this characteristic histologic feature cannot be seen consistently (13). As in our patient, dermoscopy of developed lesions reveal a central porcelain-white structureless area and a rim of hairpin-like capillaries (14).

Currently, there is no consensus on the optimal treatment of MAP patients. Some case studies suggest that drugs like aspirin, pentoxifylline, heparin, and dipyridamole maybe effective for cutaneous lesions of MAP (12). Interestingly, eculizumab (an anti-C5 monoclonal antibody) and treprostinil (a vasodilator) have also proven effective in some cases (3, 11). In all, owing to the poor prognosis of MAP, early diagnosis and timely treatment are critically needed.

## Data Availability Statement

The original contributions presented in the study are included in the article/Supplementary Material, further inquiries can be directed to the corresponding authors.

## Ethics Statement

The studies involving human participants were reviewed and approved by the Medical Technology Clinical Application Ethics Committee of The First Affiliated Hospital of Fujian (No. [2021]105). Written informed consent to participate in this study was provided by the participants' legal guardian/next of kin. Written informed consent was obtained from the individual(s) for the publication of any potentially identifiable images or data included in this article.

## Author Contributions

CJ and H-QW: conception and design. CJ: administrative support. Y-TC and YG: provision of study materials or patients. H-QW, YG, and X-PG: collection and assembly of data. H-QW and YG: manuscript writing. All authors final approval of manuscript.

## Funding

This work was supported by grants from the Natural Science Foundation of Fujian Province (No. 2020J02053).

## Conflict of Interest

The authors declare that the research was conducted in the absence of any commercial or financial relationships that could be construed as a potential conflict of interest.

## Publisher's Note

All claims expressed in this article are solely those of the authors and do not necessarily represent those of their affiliated organizations, or those of the publisher, the editors and the reviewers. Any product that may be evaluated in this article, or claim that may be made by its manufacturer, is not guaranteed or endorsed by the publisher.

## Supplementary Material

Supplementary Figure 1 A timeline of patient evaluation and care.
