# Supplementary figures and images for: Case Report: Pediatric Malignant Atrophic Papulosis With Small Bowel Perforation and Positivity of Anticardiolipin Antibody
Source: Front Pediatr. 2021 Dec 10;9:764797. doi: 10.3389/fped.2021.764797 (PMC8703221; doi:10.3389/fped.2021.764797)

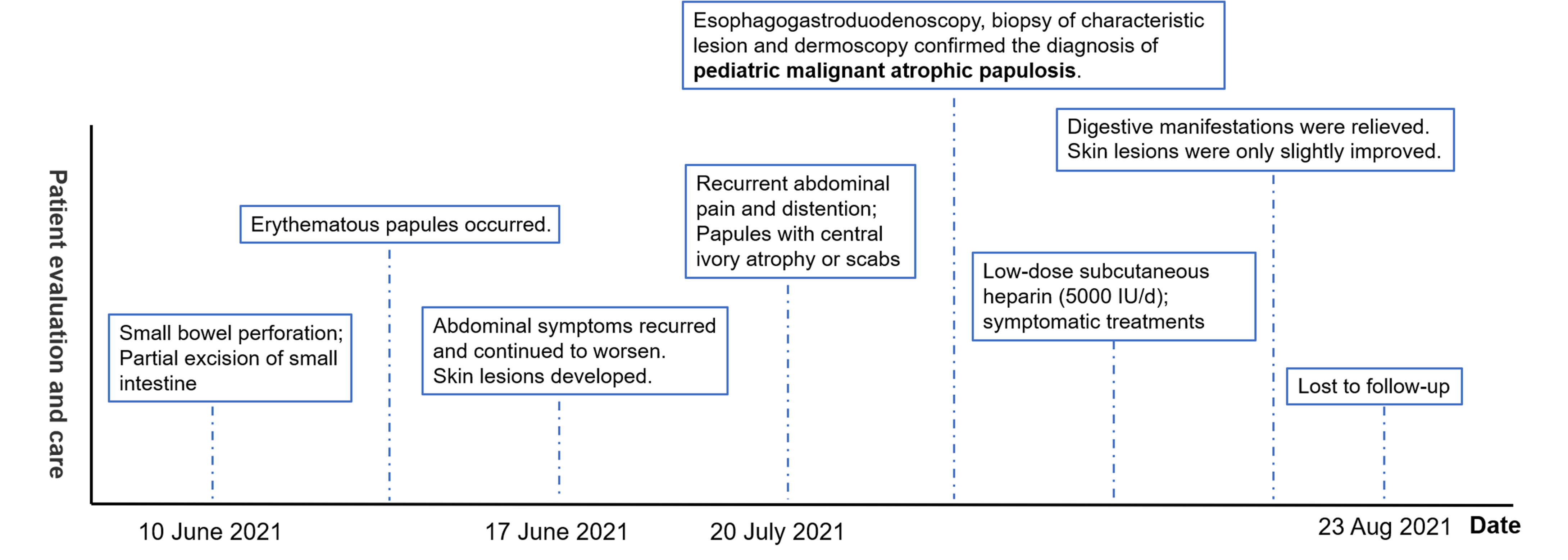

Supplement: Supplementary Figure 1 — A timeline of patient evaluation and care. [file Image_1.TIF]
